# Elucidating the Sources of *β*-Catenin Dynamics in Human Neural Progenitor Cells

**DOI:** 10.1371/journal.pone.0042792

**Published:** 2012-08-20

**Authors:** Orianne Mazemondet, Mathias John, Stefan Leye, Arndt Rolfs, Adelinde M. Uhrmacher

**Affiliations:** 1 Modelling and Simulation Group, Institute of Computer Science, University of Rostock, Rostock, Germany; 2 BioComputing Group, Lifl & Iri, University of Lille 1, Lille, France; 3 Albrecht-Kossel-Institute for Neuroregeneration (AKos), Center for Mental Health, University of Rostock, Rostock, Germany; Northwestern University Feinberg School of Medicine, United States of America

## Abstract

Human neural progenitor cells (hNPCs) form a new prospect for replacement therapies in the context of neurodegenerative diseases. The Wnt/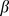-catenin signaling pathway is known to be involved in the differentiation process of hNPCs. RVM cells form a common cell model of hNPCs for *in vitro* investigation. Previous observations in RVM cells raise the question of whether observed kinetics of the Wnt/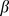-catenin pathway in later differentiation phases are subject to self-induced signaling. However, a concern when investigating RVM cells is that experimental results are possibly biased by the asynchrony of cells w.r.t. the cell cycle.

In this paper, we present, based on experimental data, a computational modeling study on the Wnt/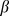-catenin signaling pathway in RVM cell populations asynchronously distributed w.r.t. to their cell cycle phases. Therefore, we derive a stochastic model of the pathway in single cells from the reference model in literature and extend it by means of cell populations and cell cycle asynchrony. Based on this, we show that the impact of the cell cycle asynchrony on wet-lab results that average over cell populations is negligible. We then further extend our model and the thus-obtained simulation results provide additional evidence that self-induced Wnt signaling occurs in RVM cells. We further report on significant stochastic effects that directly result from model parameters provided in literature and contradict experimental observations.

## Introduction

Human neural progenitor cells (hNPCs) potentially form a new basis for the *in vitro* growing of neuron populations that can be used for replacement therapies in the context of neurodegenerative diseases, such as Parkinson's or Huntington's diseases [1], [2]. They undergo the processes of proliferation, i.e., successive cell division controlled by the cell cycle, and differentiation into neural cells, i.e., neurons and glial cells. In order to deploy hNPCs for replacement therapies, a clear understanding of their proliferation and differentiation processes is essential.

ReNcell VM cells (RVM cells) derived from the ventral midbrain of a ten week old fetus [3] form an appropriate cell model for the *in vitro* study of hNPCs differentiation: they stay in proliferation as long as growth factors are present and differentiate into neurons and glial cells after growth factors removal [4]–[6].

The Wnt/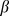-catenin signaling pathway is known to be involved in the proliferation and differentiation processes of neural cells [7]–[9], particularly in those of RVM cells [10]–[12]. It denotes a cascade of reactions that is induced by extracellular Wnt molecules at the cell membrane and that leads to an accumulation of 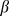-catenin in the cytosol. Consecutively, 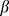-catenin is relocated to the nucleus where it activates the transcription of genes including the gene encoding for Axin protein. In this way, a negative feedback loop is established, since Axin forms the major component of the 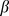-catenin destruction complex assembling in the cytosol [13].

Our previous *in vitro* analyses [12] show that Wnt signaling pathway is active during the early differentiation (first 6 hours) of RVM cells and suggest that Wnt molecules are expressed by RVM cells themselves, i.e., self-induced Wnt signaling. That is, cells secrete Wnt molecules without any exogenous stimulus but only due to the growth factor removal that induces the differentiation process. Self-induced Wnt signaling occurs in embryonic stem cells [14], both in an autocrine (cells signal to themselves) and paracrine (signaling to neighbor cells) fashion. Autocrine Wnt/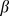-catenin signaling has been shown to occur in neural stem cells [15] and in brain development [16], [17] but not in hNPCs, in particular. Evidence for self-induced signaling in RVM cells are: endogenous expression of Wnt ligands and signaling proteins, as well as spatio-temporal traffic of the pathway signaling proteins, in both cases without addition of external Wnt signal [12]. Mainly, the two hallmarks of the pathway activation: expression of Axin gene and cytosolic accumulation of 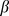-catenin, have also been observed.

Investigations of RVM cells *in vitro* are hampered by the heterogeneity of cell populations w.r.t. cell cycle states. That is, cells that are in phases S, G2, or M, rather than G1, cannot adapt to growth factor withdrawal right away. Thus, only a fraction of a RVM cell population starts differentiation immediately [12]. This asynchrony may bias the results of experimental work. For the time being, techniques to synchronize RVM cell populations during proliferation could not be successfully applied.

Computational modeling provides a way to circumvent the limitations of wet-lab experiments. The basic idea is to create an abstract representation of the system under study, a formal model, which is then analyzed with the help of computers. Models to describe a system's dynamics are in need of kinetic parameters, such as rate constants. The closer kinetic parameters relate to experimental data the more reliable the results of a modeling study are.

Stochastic modeling, as described in [18], considers models in terms of chemical reactions and multisets of molecules, which represent chemical solutions. Molecular interactions are regarded as discrete events randomly distributed in time. Analysis of stochastic models in terms of stochastic simulation provides distinct sequences of molecular interactions, with each simulation run being a different sequence. Stochastic effects have been shown to have significant impact on the dynamics of biochemical systems, especially in signaling pathways where key players appear in relatively low abundance [19]. Spatial aspects, such as molecular location or crowding, may add to this [20].

Deterministic modeling studies usually transform chemical reactions into ordinary differential equations (ODEs) and regard concentrations instead of multisets of molecules. The equivalent to simulation in the context of ODEs is numerical integration. Studies based on ODEs form an approximation of the stochastic approach that neglects the stochastic effects and thus may miss significant variations in the dynamics of systems [21], [22]. Furthermore, models expressed in ODEs often largely abstract from chemical reactions by aggregating several chemical species and reactions, e.g., in order to deal with a lack of kinetic parameters. This may largely hamper the switch back to the stochastic realm.

In this paper, we present, based on experimental data, a computational modeling study on cell cycle asynchrony and self-induced signaling in the context of the Wnt/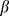-catenin pathway in RVM cells. Therefore, we derive a model of the core components of the Wnt/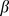-catenin pathway from the reference model of this pathway in *Xenopus* oocyte (referred to as the *Lee model* subsequently) [23] and validate it with experimental data for RVM cells, as obtained in our prior work [12]. Our model extends on the *Lee model* as it is fully specified in terms of chemical reactions, which allows us to easily switch between the stochastic and deterministic domain. Furthermore, it covers spatial aspects w.r.t. molecule location in compartments. For this, we provide additional experimental data on compartment volumes and molecule distribution in space.

We extend this core model with means of cell populations and cell cycle asynchrony based partly on our own experimental data and partly on data from the literature for the distribution of RVM cell populations over cell cycle states. We show, based on the comparison of experimental *in vitro* and *in silico* simulation data, that the impact of the cell cycle asynchrony on the average 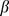-catenin dynamics in cell populations as observed in wet-lab experiments is negligible. We additionally extend our model with mechanisms for self-induced signaling. Comparing further results from simulation studies to experimental data allows us to provide additional evidence that self-induced Wnt signaling may occur in RVM cells. Moreover, we show that in our model of RVM cells, low Axin amounts, such as suggested by [23] for *Xenopus* oocyte, lead to significant stochastic effects that contradict experimental observations and that are not observable in deterministic investigations. To the best of our knowledge, so far, no results on stochastic investigations have been presented in the context of the Wnt/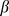-catenin pathway.

### Related work

The reference model of the Wnt/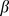-catenin pathway, the *Lee model*, is based on ordinary differential equations (ODEs) [23]. It represents the pathway in the *Xenopus* oocyte and is based on experimental data. Model analysis revealed that Axin is the limiting protein of the system due to its low abundance. Furthermore, Axin turnover strongly regulates 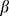-catenin dynamics.

Three follow-ups of the *Lee model* exist. The work presented in [24] and [25] extend the *Lee model* with one and two negative feedback loops, respectively. The latter shows that the addition of the negative feedbacks leads to an adaptation of the parameters given in the *Lee model*, especially an increase of 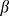-catenin and Axin turnover. The work presented in [26] aims to reduce the *Lee model*.

A first study of 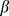-catenin's roles in correlation with its cellular location is presented in [27]. Based on the *Lee model*, they analyze, in the context of colorectal cancer, the balance between the two roles of 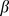-catenin: its participation in cell adhesion at the membrane and in the Wnt pathway in the cytosol. However, their computational model only considers single cells and does not take into account compartments such as the cytosol or the membrane.

An exhaustive review of models related to the Wnt pathway is available in [28]. It covers models of the Wnt pathways and their implications in animal development and in cross-talks.

## Results and Discussion

In the following, we first recapitulate the experimental data resulting from our prior work [12]. They form the crucial basis of any conclusion drawn in this study as they are the reference to which we compare our simulation results of 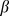-catenin dynamics in RVM cells. Then, we describe our core model of the Wnt/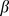-catenin pathway and its evaluation to experimental and literature data. This is followed by a report on the stochastic effects on our model's behavior. Subsequently, we discuss our investigations on cell cycle asynchrony in the context of the Wnt/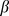-catenin pathway and on self-induced Wnt signaling in RVM cells.

### Experimental data on 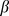-catenin dynamics in RVM cells

**Figure 1:**
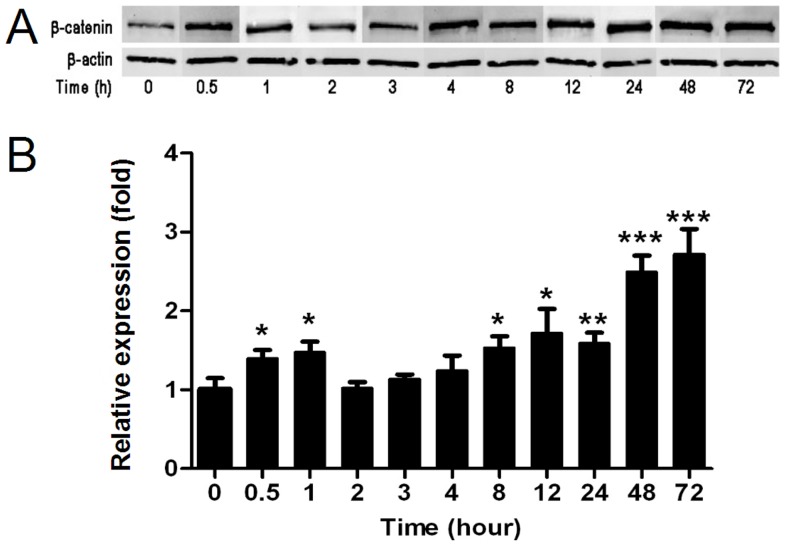
Nuclear 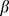-catenin during RVM cell differentiation. (A) Representative Western-blot from which the 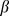-catenin protein amount was quantified (B). Time point 0 stands for control using proliferating cells and 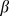-actin was used as a loading control. The signal intensities at 0 hour are normalized to 1.0 and the values are presented as mean 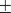 standard error on the mean from at least 3 independent experiments. The figure is reproduced from [12] (Figures 8C and 8D) where details about experimentation can be found, as well as in the section Materials & Methods.

Our study is based on wet-lab data obtained from Western blot experiments [29] presented in Figure 1, and reproduced from the Figure 8 in [12]. These data present the dynamics of nuclear 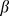-catenin in RVM cell populations from the starting point of differentiation, i.e., growth factor removal (time point 0 hour) until 72 hours after.

The data show two significant increases of 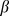-catenin during the cell differentiation. The first with a peak at ca. 1 hour and the second continuously growing from 8 hours on (Figure 1B). Such biphasic activity of Wnt is known during embryonic stem cell development [30], [31]. We expect the first increase to be a direct result of the Wnt/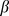-catenin pathway activity, e.g., through crosstalk with growth factor pathways [32]. The source of the second increase, however, remains controversial. On one hand it may result from cell cycle asynchrony. On the other hand, it may be caused by a subsequent self-induced signaling from time point 8 hours on, as the Wnt signal is also developmental-stage specific [30]. Whereas we show in the 3rd section of this chapter that the first hypothesis can be rejected, we provide evidence for the second in the last section of this chapter. After 24 hours, another accumulation of 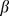-catenin can be observed. However, at that time, the cell population is already heterogeneous due to differentiation, such that the accumulation may originate from multiple sources. Therefore, our study presented focuses only on the first 12 hours.

The measurements presented in Figure 1 are in terms of relative fold changes compared to time point 0 hour, whose value is set to 1.0 (see Materials & Methods). Throughout this paper, when comparing wet-lab experiments to simulation results, we therefore consider relative amounts of nuclear 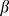-catenin (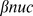 in our model), where value 1.0 represents the initial concentration or initial amount in deterministic or stochastic simulations, respectively.

### Model of the Wnt/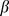-catenin pathway in RVM cells

In this section, we describe our core model and detail its underlying assumptions. We evaluate the model based on two deterministic simulation experiments, whereby the first compares to the *Lee model* to show that we cover the basic machinery of the Wnt/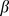-catenin pathway, as it is currently known, and the second compares to our own experimental data. Parameter sets are partly derived from literature [23], [33] and from wet-lab experiments [10], [12]. Furthermore, due to unknown parameters, each evaluation procedure involves some *in silico* experiments fitting the behavior of the model to the respective reference behaviors (parameter fitting experiments). Therefore each evaluation study comes with its individual sets of parameters. The parameter set resulting from the comparison to our own experimental data serves as a basis for the investigations as reported in the subsequent sections.

#### Model definition

**Figure 2:**
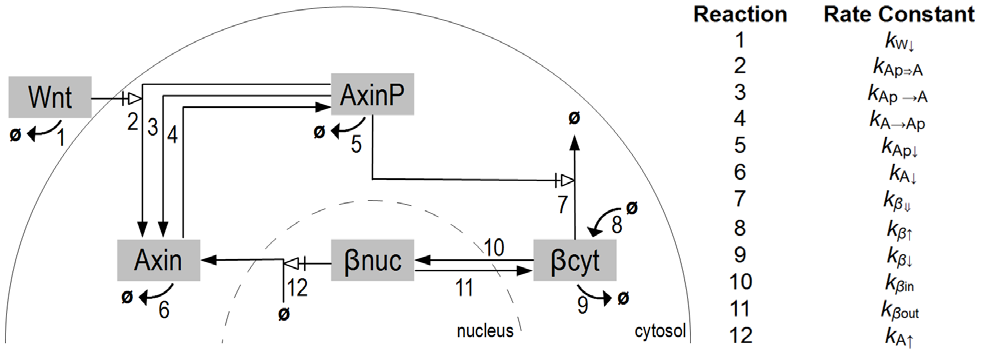
Schematic representation of the intracellular Wnt/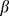-catenin pathway model. A cell is composed of two compartments, the cytosol and the nucleus, separated by the dashed lane. The five species are framed in gray. Only the species *Wnt* is extra-cellular. For a given reaction, an arrow-less lane shows the reactant(s), and an arrow lane points to the product. Bended arrows represent protein production for reaction 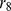 or protein decay for reactions 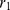, 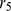, 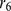, and 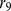. An open arrow coming after a large vertical bar represents a trigger effect (according to the Systems Biology Graphical Notation [60]) where the reactant is not consumed in the reaction, but necessary for the process to take place. The reactions' numbers correspond to the ones in Table 1.

Our core model of the Wnt/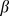-catenin pathway is derived from the *Lee model* and covers the basic components and processes in two compartments. In the cytosol, 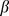-catenin is destructed by the degradation complex, which forms around Axin and is deactivated by Wnt. In the nucleus the negative feedback loop is established that consists in 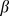-catenin triggering Axin production, leading to a higher amount of degradation complex in the cytosol. The nucleus is assumed to be spherical and the cytosol, as it is surrounding the nucleus, to form a spherical shell, see Figure 2.

We only consider the three main proteins: Wnt, 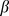-catenin, and Axin. They are represented by five species. We introduce two species for 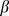-catenin, 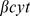 and 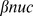, to represent its cellular location in the cytosol and in the nucleus, respectively, and two species for Axin reflecting the phosphorylation state of Axin (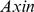 and 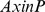). Following the ideas in [34], Axin entirely abstracts the degradation complex: 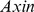 and 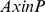 representing the inactive and active degradation complex, respectively. Axin only occurs in the cytosol. Furthermore also Wnt is located in the cytosol, since we do not consider the processes of transferring the Wnt signal from the outside to the inside of the cell.

Our model is entirely expressed in terms of chemical reactions (listed in Table 1). can either decay (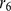, 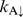) or phosphorylate to become 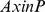 (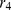, 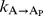). The latter can again dephosphorylate (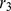, 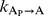), decay (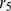, 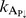), or degrade 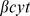 (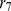, 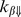). We represent the Wnt-dependent deactivation of the degradation complex by 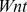 promoting 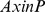 dephosphorylation (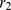, 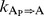). Notice that by this the amount of 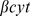 is indirectly increased. Moreover, this forms also the only way in which the Wnt signal influences 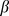-catenin dynamics.

**Table 1:** Reactions of the intracellular Wnt/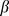-catenin pathway model.

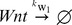	(1)
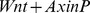 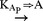 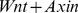	(2)
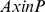 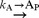 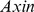	(3)
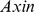 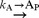 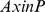	(4)
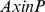 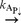 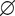	(5)
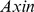 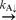 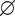	(6)
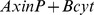 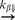 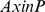	(7)
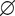 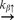 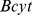	(8)
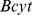 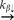 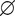	(9)
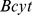 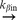 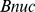	(10)
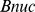 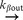 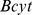	(11)
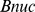 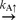 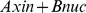	(12)

All reactions are following Mass action kinetics, but 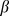-catenin production (1) that is a constant flux. The reaction numbers correspond to the ones in the model schema (Figure 1).

Previous wet-lab experiments suggest that the passage from cell proliferation to differentiation after removal of growth factors is accompanied with an extra-cellular presence of active Wnt molecules that is self-induced by the cell population [12]. Our model reflects this fact by considering a given initial amount of 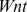. The effect of the signal decreases over time, since 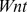 decays (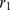, 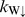) and is not further produced.

For 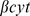, there exists a constant flux of production (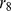, 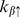) and decay (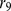, 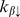). Furthermore, 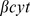 travels between the cytosol and the nucleus in both directions (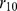, 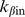, and 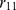, 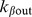). In the nucleus, 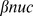 induces 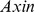 production (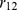, 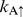), representing the pathway's negative feedback loop. Except for 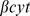 production (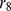, 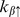) that is modeled as a constant flux, all the reactions follow Mass action kinetics.

It is worth noting that the *Lee model* contains reactions for both basal Axin and basal 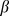-catenin production. By contrast, our model only contains basal 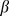-catenin production. The reason is that basal Axin production is a constant flux, i.e., its reaction speed is independent of species concentration levels. As the reaction rate constant of this flux is, according to the *Lee model*, five orders of magnitudes lower than the one of basal 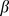-catenin production, its impact is negligible and can therefore be omitted.

#### Model assumptions

We adopt the usual assumptions made when modeling cell-biological systems, i.e., constant compartment volumes, molecules without volumes, etc. [18]. Besides focussing only on the major components of the Wnt/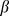-catenin pathway, we also neglect possible crosstalks with other pathways, such as Ryk [35] or Notch [36]. Following the ideas in [34], we abstract the degradation complex by only one of its components, Axin. This is possible since Axin is the limiting factor of the degradation complex due to its low amount in comparison to the other three components, GSK3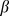, APC, and CKI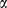 [23]. The binding of Wnt molecules to the membrane receptors is not represented as it still remains, biologically, poorly understood. The reaction of *Wnt decay* (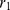) represents both its consumption and deactivation after signaling. The nucleo-cytoplasmic shuttling of 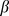-catenin remains yet unclear [37], thus we introduce the motion of 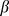-catenin as a simple diffusion [38] with rate constants based on experimental data [33].

#### Model evaluation by comparison to the *Lee model*

In the following, we provide an evaluation study for the overall concept of our model, with its reactions and the assumptions made, based on a comparison to the *Lee model*. It focuses on comparing the concentration of 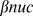 to the concentration of free 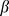-catenin in the *Lee model* (that considers only the cytosolic compartment).

In our model, initial concentrations are taken from the *Lee model*, as they were retrieved from experiments in *Xenopus* oocyte. An exception forms the concentration of *Wnt*. In the *Lee model*, Wnt molecules are not considered but replaced by a rather abstract signal called 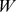. The signal 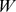 has a real value between 0 and 1 that decreases exponentially following: 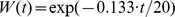. In our model, Wnt molecules decay over time following a Mass action kinetics (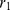). The kinetic rate value for this reaction is obtained from the previous function 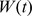: 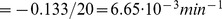, and an initial Wnt concentration of 100 (unitless) is assumed. Reactions for decay and production and for Axin decay are conserved from the *Lee model*. Table 2 presents the parameter names and values as used in the *Lee model* and their respective names in our model.

**Table 2:** Parameters from the *Lee model* [23] used in our model and their stochastic conversion.

*Lee model*		*our model*	
Parameters	Deterministic values	Parameters	Stochastic values
***Species initial values***			
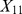	25.01 nM	n*βcyt*	13141 molecules
		n*βnuc*	5347 molecules
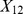	0.02 nM	n*Axin*	11 molecules
		n*AxinP*	11 molecules
W	0 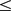 W 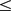 1	n*Wnt*	100 (dimensionless)
***Rate constants***			
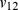	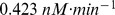	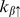	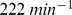
W(t)	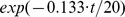	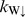	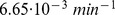
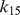	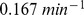	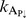	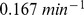
		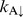	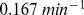
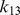	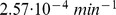	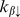	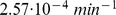

Parameters' names and values are given as in [23]. Their corresponding names in our model are given with their stochastic values.

Table 3, Set 1 provides the values of all model parameters: The kinetic rates of reactions 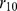 and 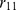 are taken from literature [33]. As several other rate constants are unknown, we performed parameter fitting experiments (parameter estimation details are given in Materials & Methods Section).

**Table 3:** List of model parameters.

Parameters	Deterministic values		Stochastic values	
	Set 1	Set 2	Set 3	Set4
*Species initial values*				
n*βcyt*	24.9 nM	23.6 nM	11145 molecules	12989 molecules
n*βnuc*	24.9 nM	23.6 nM	4532 molecules	5282 molecules
n*Axin*	0.007 nM	0.051 nM	144 molecules	252 molecules
n*AxinP*	0.042 nM	0 nM	125 molecules	219 molecules
n*Wnt*	100	1000	1000	1000
*Rate constants*				
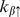	0.232*	0.571*	420	600
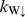	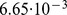	0.27	0.6	0.27
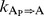	7	10	10	20
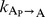	0.3	0.0182	0.03	0.03
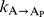	3	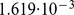	0.03	0.03
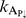	0.167	0.167	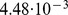	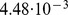
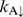	0.367	0.0167	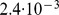	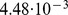
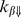	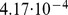 *	52.5*	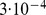	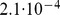
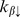	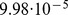	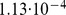	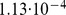	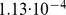
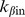	0.0549	0.0549	0.0549	0.0549
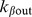	0.135	0.135	0.135	0.135
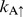	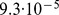	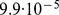	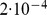	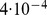

Parameters used in our model for the different *in silico* experiments. Sets 1 and 2 are the results of the parameter fitting experiments comparing to the *Lee model* and our experimental data, respectively. Sets 3 and 4 are obtained from our stochastic and our cell cycle investigation, respectively. All rate constants are in 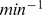 but the ones indicated by “*”: the deterministic values of 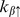 are given in 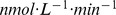, and the deterministic values of 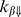 are given in 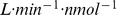.

**Figure 3:**
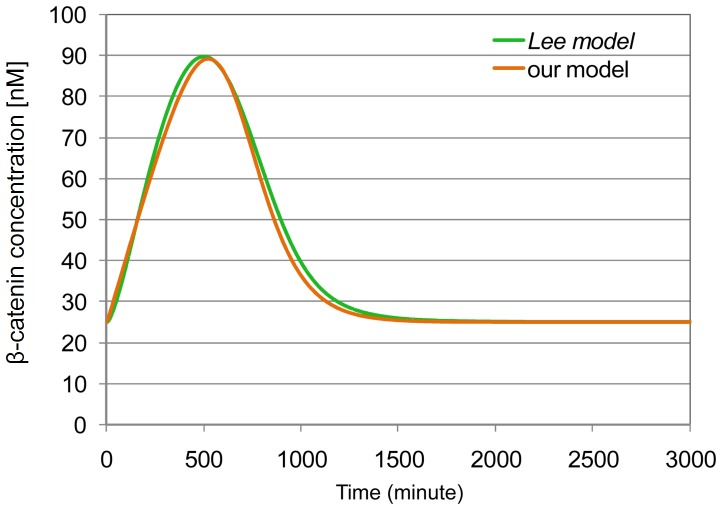
Comparison of 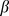-catenin dynamics between our core model and the *Lee model*. Time-dependent response of 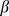-catenin under transient Wnt signal (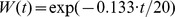 introduced into the system at the steady-state, at 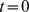). The dynamics of total in the and of nuclear 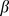-catenin (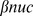) in our core model are corresponding. Simulations of the *Lee model* are performed through the JWS platform [55].

The results of simulation experiments with *Wnt* set to 100 (transient Wnt stimulation) in our model, and 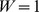 in the *Lee model* are provided in Figures 3. They show a good fit between the concentrations of 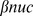 in our model and 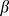-catenin in the *Lee model*.

#### Model evaluation by comparison to experimental data

Here, we present the results of a parameter fitting experiment that compares the dynamics of in 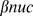 our model to those provided by our wet-lab experiments (see Figure 1). Only the first increase in the data is subject to the fitting, since it is the one considered to reflect the dynamics of the Wnt/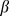-catenin pathway as initially induced by growth factor removal. Notice that at this point we compare single *in silico* cells to the average behavior of entire populations as measured in our wet-lab experiments. This means in particular that we implicitly assume a synchronized RVM cell population w.r.t. the cell cycle.

The results of our experiment are provided in Table 3, Set 2. Whereas initial species values remain as before, the values of most rate constants are adjusted. In particular, the value of the rate constant of Axin-dependent 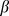-catenin degradation (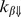) shows a significant increase (see the section on stochastic investigations for further details).

**Figure 4:**
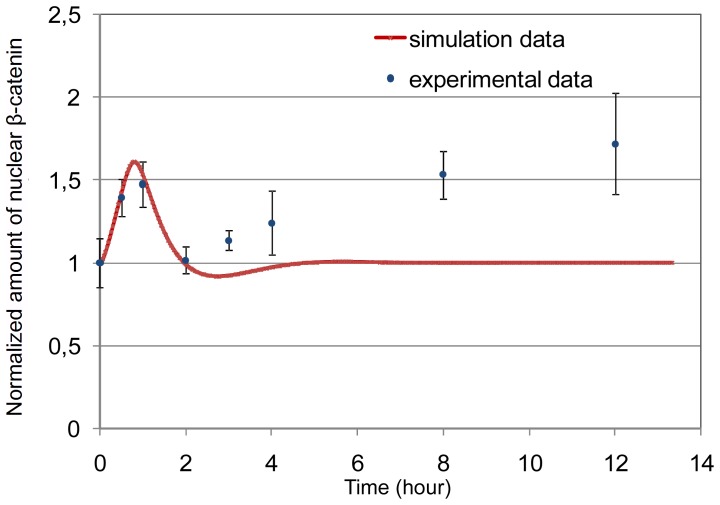
Model evaluation in comparison to the experimental data. Comparison of the model time course to the experimental data for nuclear 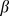-catenin dynamics. The simulation data fit the experimental ones for the first 2 hours.

In Figure 4, results of simulation experiments with the obtained parameters are provided. We obtain a good fit to the first increase in our data. Moreover, after two hours of differentiation, 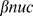 remains constant and no second increase can be observed.

### Stochastic effects due to low AxinP amounts

In this section, we report on stochastic fluctuations in the dynamics of our model that contradict our wet-lab observations. We show that these fluctuations directly result from the low Axin amounts as suggested by the *Lee model* [23] rather than from our specific choices of parameter values. This study requires a transformation of the concentrations and kinetic rate constants in Table 3 (Set 2) into molecule numbers and stochastic rate constants, following, e.g. [39], (Table 3, Set 3).

**Figure 5:**
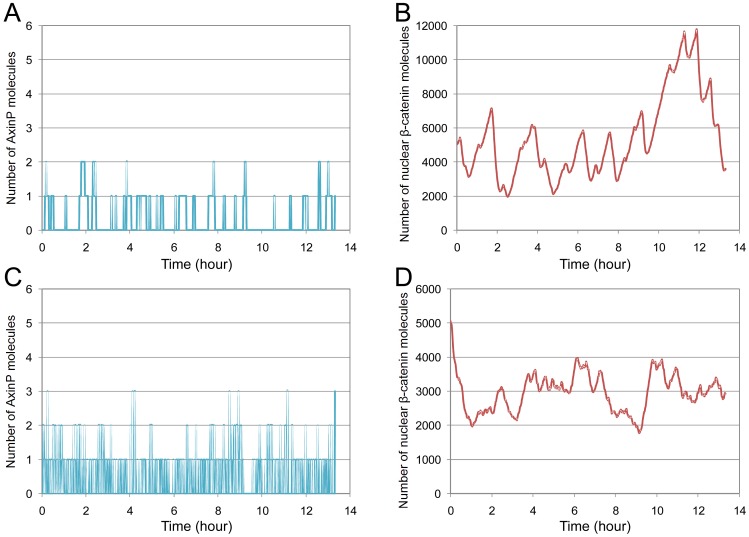
Effects of AxinP on 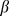-catenin dynamics according to stochastic simulation. In absence of Wnt signal, small variations of 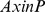 (A) influence 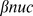 dynamics (B) leading to high variance. Increase of 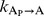 and 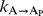 by 1000 accelerate 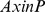 changes (C), but 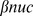 fluctuations still remain too high (D). Each plot represents a single simulation run.

The stochastic fluctuations in the 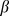-catenin dynamics resulting from Parameter Set 3 are shown in Figures 5A, 5B. These figures present the amount of 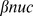 and 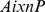 molecules over time in absence of Wnt signal, as the result of a single simulation run. Large fluctuations in the 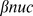 amounts can be observed that were not visible in the deterministic investigations of the previous section. Along with the fluctuations, we see only small differences in the number of 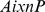, which underlines the high impact of 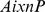 changes on the 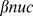 dynamics. This impact results from a comparatively high rate of 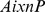-dependent 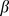-catenin degradation (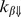) that is necessary to fit our experimental data. The stochastic fluctuations contradict our *in vitro* results, since they do not allow for a clear transient peak of 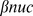 in response to the Wnt signal (not shown).

In order to exclude that these stochastic fluctuations are the result of our specific choice of parameter values, we explored different strategies to change parameter values in order to lower stochastic effects and at the same time to maintain the amounts of 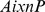 and the average behavior of the model w.r.t. 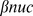 dynamics.

An obvious solution would be to decrease the rate constant of 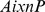-dependent 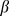-catenin degradation (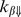) and maintaining the 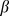-catenin level by simultaneously decreasing the flux of 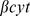 production (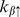). However, simulation experiments showed that already through small changes of this sort, the amount of 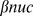 is prevented from increasing in response to Wnt signal (results not shown).

Only one other parameter modification strategy seems to be plausible to us: to deploy the inertia of 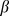-catenin reacting on changes in 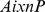 amounts. Since the amount of 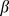-catenin is relatively high, observable changes due to differences in 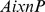 amounts can only take place with a certain delay. The amount of 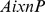 is impacted only by its decay (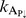) and its de-/phosphorylation (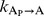, 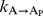). Instead of increasing one of the corresponding rate constants separately, which would result in a change in the overall amount of 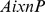, one can increase the rate constants for de-/phosphorylation simultaneously. Figures 5C, 5D show the results when increasing the rate constants for dephosphorylation (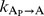) by about 1000 times and the one for phosphorylation (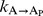) accordingly. Changes in 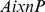 numbers happen much faster. However, although they are lower, the stochastic fluctuations in the 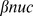 dynamics are still too high. Raising the dephosphorylation rate (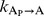) even more seems not plausible to us, since then changes on the few 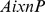 molecules happen in periods of milliseconds.

We are therefore convinced that in our model with low 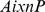 level, as derived from the *Lee model*, stochastic fluctuations in the 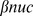 level cannot be limited to fit our experimental data. As additional support for this statement, we also provide an Sbml version of our model in the supplementary material (Model S1).

Our results may indicate on one hand that our RVM cells contain higher amounts of Axin than suggested for the *Xenopus* oocyte by [23]. This is supported by the recent work of Tan and colleagues [40] as they report that mammalian cells have a higher Axin concentration than the *Xenopus* extract and that yet 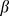-catenin concentrations remain higher than Axin concentration. Notice, however, that in prior wet-lab experiments, we failed to detect Axin. On the other hand, our model and thus our understanding of the system may miss an important mechanism to reduce stochastic noise, such as dimerization [41], [42] or additional feedback loops [43].

**Figure 6:**
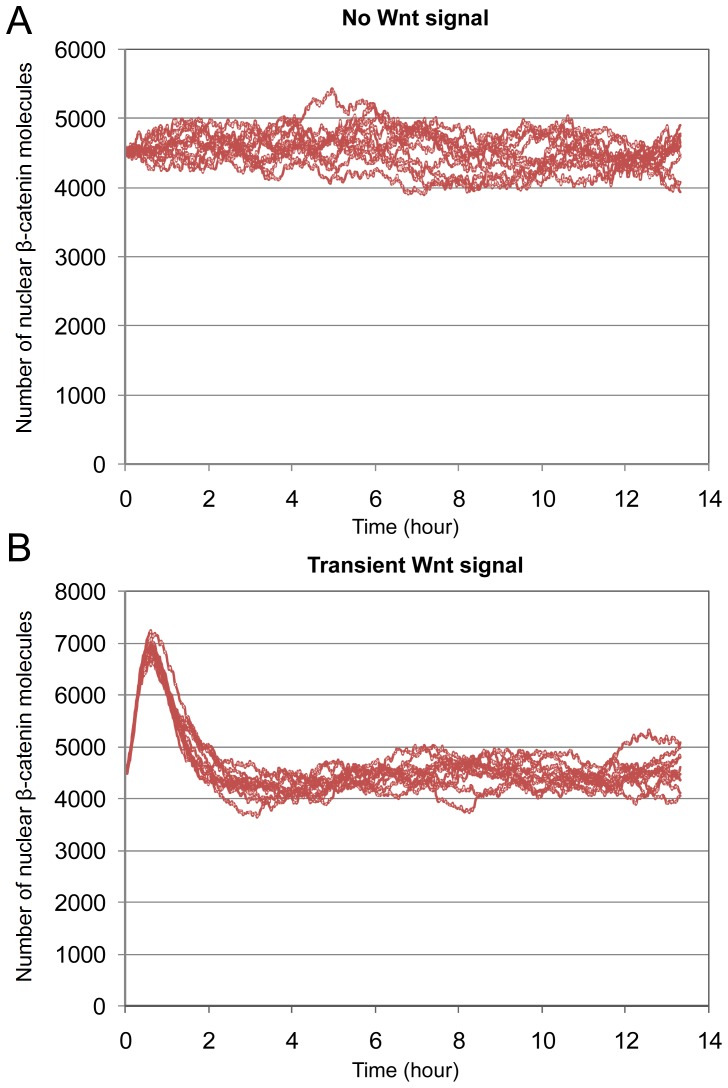
Nuclear 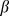-catenin dynamics with higher number of AxinP. (A) In absence of Wnt signal. (B) Under transient Wnt signal. The plots represent 10 simulation runs.

As a basis for our subsequent investigations, we performed additional parameter fitting experiments, thereby stepwise increasing the amount of 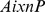 and 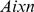. As a result, we found a parameter set with acceptable stochastic fluctuations at an initial amount of 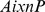 of 125, see Table 3, Set 3. In Figure 6A, the dynamics of 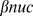 for 10 simulation runs are shown, where the Wnt signal is switched off (*Wnt* = 0). One can see that the fluctuations in 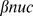 levels have been effectively decreased. The t-test provides that at the time point of maximum deviation (ca. 426 minutes), the mean value of 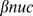 numbers in the 10 simulation runs lies in the interval of 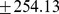 (ca. 5.5% of the simulated mean value) around the simulated mean value with a confidence of 95%. Figure 6B shows the dynamics of 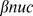 for 10 simulation runs when the Wnt signal is switched on. We observe in our simulation a transient peak within the standard deviation of the experimental data.

### Impact of the Cell Cycle on 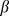-catenin dynamics in RVM cells

In this section, we show that in the results of wet-lab experiments, the impact of the cell cycle on the average dynamics of 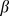-catenin in RVM cell populations can be neglected.

The idea of extending our model to represent asynchronous cell populations is to consider a number of copies of our core model, each representing an individual cell. Following [12], cells have to first complete the cell cycle before they can process the Wnt signal. As for our previous model *Wnt* is not produced and decays overtime. However, we assign an individual delay to the start time of Wnt degradation (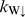) and Wnt-dependent Axin dephosphorylation (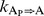) of each cell that represents the cell delay to exit the cell cycle. That is the corresponding reactions 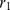 and 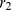 are ensured not to happen before a fixed time point has passed. Cells, which are already in the G1 phase at the beginning of a simulation experiment, i.e., at time point 0 hour, start to perform their reactions without any delay. Notice that, since each cell has its own pool of Wnt and also reaction 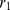 is delayed for each cell individually, we assume self-induced signaling to happen in an autocrine fashion. The integration of delays into our stochastic model is described in the Materials & Methods Section.

Cell cycle states are assigned to cells following the distribution of cells over the cycle phases as obtained from experiments (see Figure 2 in [12] and details in Materials & Methods). For example, in a population of 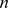 cells, 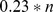 cells are assigned to state 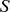. Potential rounding problems are solved by randomly assigning states to remaining cells with probabilities to be in certain states following our experimental data. Notice that we do not separate G2 and M phases. The delay for each cell is computed in the following way: cells are assumed to be equally distributed over their respective states. That is, to a cell in state 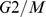 a delay 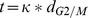 is assigned, where 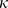 is equally distributed in 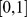 and 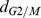 is the duration of phase 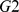. Similarly, since each cell in state 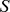 has to additionally pass state 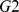, the delay of a cell in state 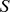 is given by 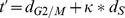, with 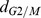 being the duration of phase 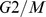. The duration of each cell cycle phase is obtained from literature [10], [44] (details in Materials & Methods).

**Figure 7:**
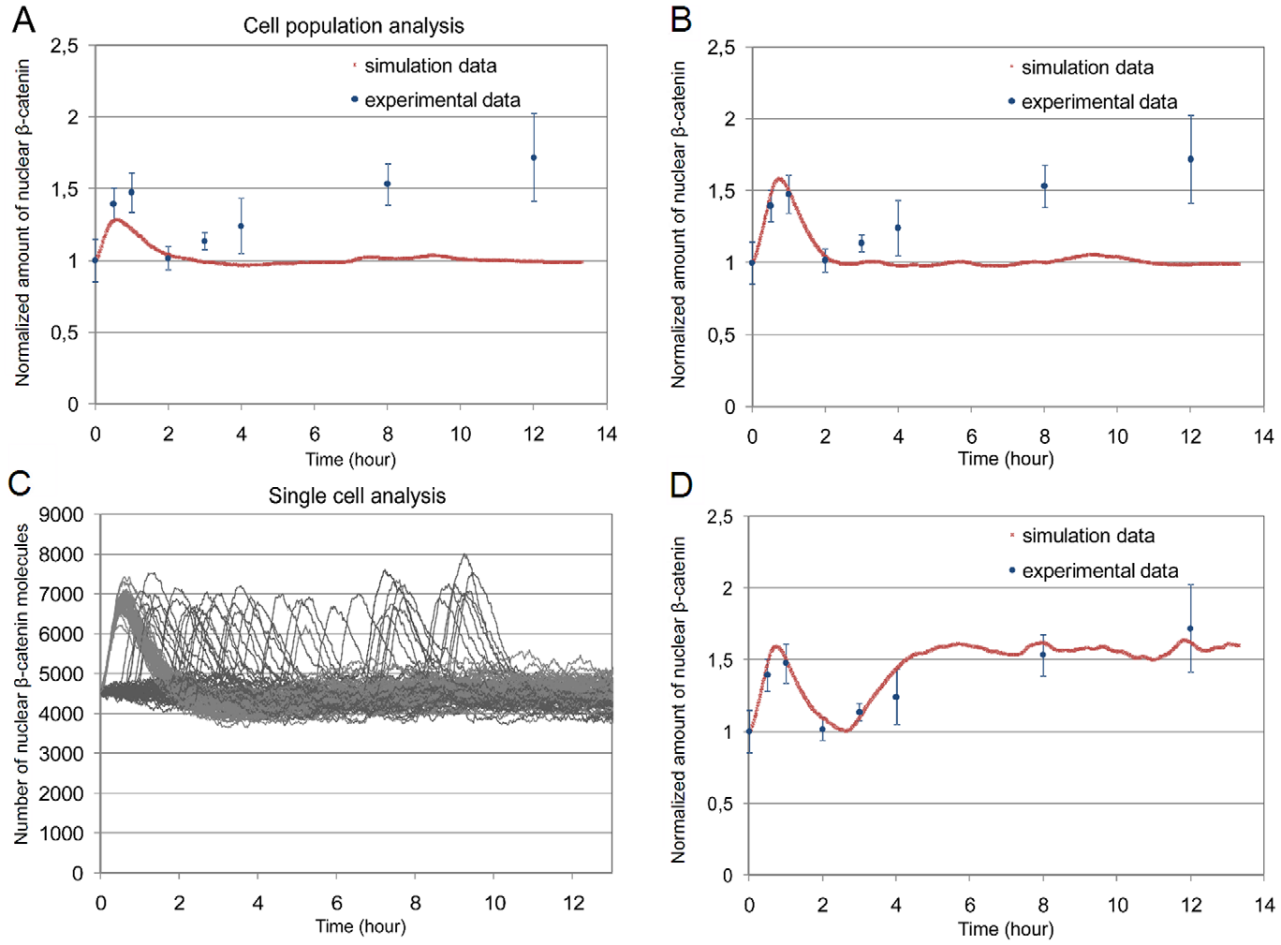
Dynamics of nuclear 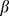-catenin in heterogeneous cell population. A population of 100 cells is simulated. (A, B, and D) represent the sum of nuclear in 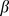-catenin 100 cells as compared to the experimental data. (C) represents the number of nuclear 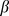-catenin in each cell individually for a population of 100 cells. (A) The total cell population is analyzed. The simulation data do not correlate with the experimental ones. (B) The cell population asynchrony toward the cell cycle does not give rise to a second increase of nuclear 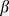-catenin after 2 hours. (C) Single cell analysis shows the delay in 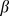-catenin dynamics due to the cell cycle asynchrony. (D) The addition of a continuous and autocrine Wnt signal to the previous experimental setting produces simulation data in perfect agreement to the experimental ones. (A, C) Parameters used are given in Table 3 (Set 3). (B, D) Parameters used for both experiments are given in Table 3 (Set 4).

We performed simulation experiments with the parameters in Table 3, Set 3, as obtained from our stochastic investigation. In Figure 7A, the sum of the number of 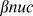 (nuclear 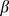-catenin) for a single simulation run with 100 cells is presented. Similar to the results of the previous section (Figure 6B), we observe a single transient increase. This, however, shows only a value about 1.28 instead of 1.48, i.e., we obtain only 86% of the expected amount. The reason is that, following our experimental data on the cell cycle in RVM cells, the 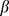-catenin amounts in most of the cells initially dedicated to the cell cycle, does not start to increase before 30 minutes, i.e., after the time of the first peak is over. Thus, only about 60% of the cells contribute to the first peak, resulting in the limited increase.

As mentioned earlier fitting experiments to obtain the parameters (Table 3, Set 3) implicitly assume that cells are homogeneously distributed over the cell cycle as we compared the behavior of single cells, i.e., one instance of our core model, with the average behavior of entire cell populations as observed in experiments. Thus, the results of our simulation experiments imply that this assumption is not feasible and that when modeling the Wnt/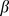-catenin pathway in RVM cells, the cells' commitment to the cell cycle may need to be considered.

We present an additional parameter set to fit the first increase in our experimental data with the simulation data in *asynchronous* cell populations (see Table 3, set 4). Only a few parameters are adjusted, as compared to the previous one. Corresponding simulation results are presented in Figure 7B. It can be seen that with the new parameter set, the first increase of nuclear 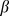-catenin is well-fitted. Furthermore, no other increase than the first one can be observed in our simulation results.

Our simulation results indicate that when studying the Wnt pathway in RVM cell populations by wet-lab experiments the impact of the cell cycle on the average 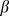-catenin dynamics can be neglected. No significant changes occur other than the increase caused by Wnt pathway activation. In particular, the second increase in the experimental data shows not to be a result of the cells' asynchrony. The reason for this becomes obvious when looking at the dynamics of 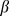-catenin for each cell separately, as presented in Figure 7C. Although, all the cells, which are initially dedicated to the cell cycle, start their 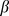-catenin increase comparatively late, they do not perform it simultaneously but rather widely distributed over time (between 0.5 and 10 hours). Thus, summing up over all cells, the individual peaks only lead to small deviations. It is important to notice here that this observation is rather independent from the particular design decisions of our model but results mainly from the distribution of RVM cells over the cell cycle as it is obtained experimentally and from the basic knowledge about the Wnt/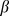-catenin pathway as represented in our model.

### Self-induced Wnt signaling

In this section, we provide new evidence for the hypothesis that the 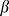-catenin dynamics from 8 hours on after the start of differentiation arises from a second wave of Wnt signal that could be self-induced. This hypothesis is in the lines with our previous study [12] listing various indicators from wet lab experiments for late self-induced Wnt signaling. Among these are an increase of Axin, Wnt ligands, and receptor gene expression, as well as an accumulation of the pathway's intracellular proteins during cell differentiation without addition of external Wnt signal (see also this article's introduction). Notice that the goal here is not to explain the detailed underlying mechanisms of self-induced Wnt signaling, but rather to explore whether our experimental data actually suggest that such a process may occur in RVM cell populations. In particular, we leave possible spatial extensions to our model to distinct autocrine from paracrine signaling as subject to future work and consider only autocrine signaling here.

To model self-induced Wnt signaling, we extend our model with a single reaction of Wnt production representing the overall process in a very abstract way. This reaction occurs with a given delay after cells exit the cell cycle. The delay is to reflect the time necessary to induce the signal. It is implemented in the same way as the one for the cell cycle reaction (details in Materials & Methods section). Once the delay is over, cells continuously produce Wnt molecules with rate constant 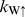 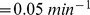, each for themselves (autocrine signaling).

We performed a simulation experiment with a cell population of 100 cells and a Wnt induction delay of 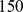 minutes (2.5 hours). Such value for the delay is plausible, since our Wnt-producing reaction encompasses various biological processes, i.e., gene transcription of Wnt molecules, their subsequent intracellular trafficking, post-translational modifications [45], secretion of Wnt in the extracellular environment, and its binding to the cell receptors.

In Figure 7D, the results of a single simulation run are presented. The first peak of 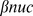 is still fitting the experimental data. Furthermore, the simulation data at the other time points, i.e., 3, 4, 8, and 12 hours, are also fitting the experimental ones. Since the previous variants of the model failed to reproduce the biphasic kinetics of nuclear 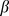-catenin, our results suggest that self-induced Wnt signaling is responsible of such dynamics and occurs in RVM cells during differentiation from 2.5 to 12 hours.

It should be noticed here that the investigations of Wnt production in RVM cells at 3, 6, and 24 hours of differentiation shows only for the latter time point an increase of Wnt mRNA level (mRNA of Wnt5a and Wnt7a) [11], [12]. These observations are, however, not necessarily in contradiction to the hypothesis of self-induced signaling whitin the first hours of cell differentiation. On one hand, without measurements of intermediate time points, Wnt production could start as soon as 8 hours. On the other hand, even without an increase in the Wnt mRNA level, self-induced signaling could occur. It is known that Wnt molecules are produced in the cell and are subsequently stored in cytosolic vesicles that can then fuse with the membrane in order to release Wnt molecules outside of the cell, inducing signaling [46]. Thus, the self-induced Wnt signaling can result not only from a constant production and secretion during RVM cell differentiation but also from an anticipated production of Wnt molecules followed by a vesicle storage and a delayed continuous secretion during differentiation. Clarification on this point could be achieved by further *in vitro* experiments, based, e.g., on a constant inhibition of the Wnt/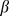-catenin pathway, in particular with Dickkopf 1, Mesd, or Porcupin [46]–[48], followed by kinetic analysis of nuclear 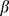-catenin between 2 and 12 hours of differentiation.

Furthermore, the occurrence of crosstalk remains to be a valid alternative to the hypothesis of self-induced Wnt signaling. Previous works show that during nervous system development [49], [50], although not during neural differentiation in particular, crosstalks occur between the Wnt/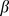-catenin pathway and other pathways, among which are the Ryk [35], Notch [36], and extracellular signal regulated-kinase (ERK) pathways [51]. Therefore, to finally answer the question of self-induced Wnt signaling in human neural progenitor cells, further *in vitro* or *in silico* investigations are required.

## Materials and Methods

### 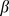-catenin Western-blotting

Western-blots were performed according to the protocol described previously in [10]. Visualization and quantification of 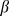-catenin were performed using the Odyssey Infrared Imaging System (LI-COR Biosciences GmbH, Bad Homburg, Germany). For the purpose of quantification, time-series samples were loaded onto the gel in a randomized and non-chronological order to reduce systematic errors [52]. Expression of 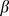-actin protein was used as a loading control to normalize the expression of 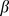-catenin. Thereby relative expression levels of 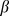-catenin proteins were determined. For each quantified Western-blot, the relative expression at a given time point is normalized to the mean of the time series. Normalization is performed for each experiment separately, as the raw data obtained for each Western-blot are not absolute values. For each time point, the mean of all experiments is computed. Thus, the values observed in Figure 1 show the mean of 26 experiments (9 cell cultures with 2 to 3 Western-blots performed for each cell culture) for the time points 0, 0.5, 1, 3, 8, 24, and 48 hours, and the mean of 11 experiments (4 cell cultures with 2 to 3 Western-blots performed for each cell culture) for the other time points 2, 4, 12, and 72 hours. Statistical evaluation was carried out using the Mann-Whitney test. An increase was considered to be statistically significant when the p-value 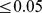 (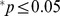, 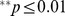, and 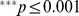) as compared to control (time point 0 hour). The values of the mean and the standard error of the mean were rescaled to the mean at time point 0 hour which is set to 1.0.

### Cell volume determination

Data on compartment volumes is a prerequisite for calculating stochastic parameters as the reaction speed is dependent on the probability of molecules to collide. As the cell volume and thus the compartment volumes might change along cell differentiation, the volume measurement is restricted to proliferating RVM cells, as at this stage their general shape is regular, following a cobble-stone pattern [3]. The global volume of proliferating RVM cells was determined using the electric cell counter CASY® (Innovatis AG, Germany) which uses impedance technique. The RVM cells were cultivated according to the protocol described previously in [10]. The cells were detached from the coated cell culture flask T75 with addition of Trypsin-Benzonase solution (2.5 ml/flask) followed by trypsin-inhibition solution (5 ml/flask). As the solutions are isotonic they do not cause cell volume changes. In presence of Trypsin, RVM cells are in nearly spherical shape within the solution suspension. This condition is necessary and sufficient for the device CASY® to retrieve the mean volume of the cells analyzed in the sample. Cell suspension (25 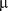
l) was diluted in 10 ml of CasyTon buffer and analyzed by CASY®. The analysis results contain the mean volume of the spherical cells passing through the capillary. Thus, given 79 cell samples, from cell passage 9 to 27, the average volume of RVM cells is 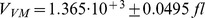. To obtain the compartment volumes, the average area of the cytosol and nucleus in proliferating cells, was measured via microscopy. The cytosol represents an average of 64% of the cell surface, whereas the nucleus represents 26% of the cell surface. These values were considered to hold true for the volume proportions, leading to:
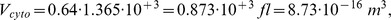


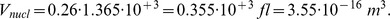



### Duration and distribution of cell cycle phases

The duration of the RVM cell cycle is given in [10] with a doubling time of 19.8
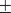
0.6 hours (ca. 1188 minutes). In order to estimate the duration of each phase, we took reference values from [44] that describes a relative duration of 50% for G1, 33.3% for S, and 16.7% for G2/M of the cell cycle entire duration in mammalian cells. Thus, the delay for the G2/M phase amounts to 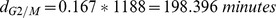 and for the S phase to 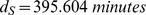. The amount of RVM cells committed in the cell cycle was retrieved experimentally as described in [12]. Around 20% of RVM cells are in G2 phase and around 23% in S phase when differentiation is induced (time point 0 hour).

### Parameter estimation & simulation

Parameter estimation for the unknown values of rate constants and species amount was done using *COPASI* [53]. The method chosen is Hooke & Jeeves [54]. Estimation experiments were supported by sensitivity analysis available in the supplementary material (Text S1). The *Lee model* simulations were performed using the online tool *JWS Online* [55]. Deterministic simulations were also done with the tool *COPASI* [53] using the deterministic LSODA method. Stochastic simulations were performed using a small extension of the stochastic simulation algorithm, the version presented in [56], that allows to directly reflect normally distributed delays (see paragraph below). We implemented this extension and performed stochastic simulation experiments based on the modeling and simulation framework JamesII [57].

### Modeling delays

We used the following approach to model delays: for each delay 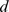 a species 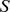 and a reaction 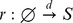 is introduced. Initially, the amount of 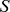 is set to 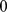. Reaction 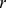 produces a single instance of 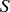 to denote the end of the delay. Reactions, which are supposed not to happen before the delay has ended, are equipped with an additional reactant and product of species 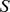, e.g., reaction 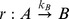 is transformed to 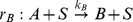. Notice that when ensuring that the amount of 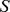 is always 1, the rate of reactions with Mass action kinetics is not affected by this modification. Reaction 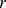 cannot be directly mapped to a stochastic reaction since it is required to happen only once and at a time point not exponentially distributed, but normally distributed in time that denotes the end of delay 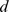. This can be achieved by using the next-reaction method [56] version of the stochastic simulation algorithm [18], which schedules the time point of each reaction in an event queue. The firing of a reaction at a fixed time point that models the end of a delay can thus be just scheduled as an additional event. We assumed that the end time point of a delay is drawn from a normal distribution on the length of the delay with a standard deviation of 5%.

Similar ideas to integrate delays into stochastic models have already been presented in [58]. Alternatively, one can also approximate events at normally distributed time points with sets of fast intermediate reactions [59].

## Supporting Information

Model S1 **We provide the details of the model in Sbml format. The parameter values correspond to the ones found in** **Table 3** **, Set 2.**
(XML)

Text S1 **We provide details regarding the parameter sensitivity analysis.**
(PDF)
