# Supplementary material for: Elucidating the Sources of β-Catenin Dynamics in Human Neural Progenitor Cells
Source: PLoS One. 2012 Aug 20;7(8):e42792. doi: 10.1371/journal.pone.0042792 (PMC3431164; doi:10.1371/journal.pone.0042792)
Supplement: Text S1 — We provide details regarding the parameter sensitivity analysis. (PDF) [file pone.0042792.s002.pdf]

Text S1 - Parameter sensitivity

Parameter sensitivity provides a measure for the impact of changes in input parameters on a model's overall behavior in terms of e.g. simulation results. It is therefore often used as a basis for parameter fitting experiments, e.g. to determine appropriate orders of magnitude for individual parameter variations, to filter those parameters that have no significant impact and are therefore less relevant for parameter fitting.

In our study, we assume the behavior of our model to be best represented by the dynamics of nuclear β-catenin (βnuc in the model), as they are also the focus of our experimental observations. For each parameter, we ran individual tests to determine the impact of changes in its value on simulation results. Our study applies to the initial parameter set (set 1) in Table 3 of the main paper. To measure the impact of a change, we compared the simulation results after modification to those with the initial parameter values. Therefore, we defined the difference between two trajectories as the Eucledian distance of value pairs at five time points (0.5, 1, 2, 8, and 12 hours) characterize β-catenin dynamics. As our study bases on stochastic simulation, runs for single parameter sets are replicated and trajectories of mean values are used for comparison. The number of runs is chosen, such that 95% of confidence for an interval of 5% at the five time points used for comparison is obtained.

The results are given in Table S1. With a precision of 5%, they show for each parameter the change of its value that is at least required for a deviation of 10% (see Formal Description below).

Table S1. Results of the sensitivity analysis.

parameter	change	parameter	change
nβnuc	0.0001	$k_{\beta\text{in}}$	0.18
n$Axin$	0.016	$k_{\text{A}\rightarrow\text{Ap}}$	0.23
n$AxinP$	0.018	$k_{\text{W}\downarrow}$	0.25
$k_{\beta\uparrow}$	0.019	$k_{\text{Ap}\Rightarrow\text{A}}$	0.28
nWnt	0.036	$k_{\text{Ap}\downarrow}$	0.37
$k_{\beta\Downarrow}$	0.04	$k_{\beta\text{out}}$	0.46
$k_{\text{Ap}\rightarrow\text{A}}$	0.042	$k_{\text{A}\uparrow}$	0.58
$k_{\text{A}\downarrow}$	0.072	$k_{\beta\downarrow}$	0.91
nβcyt	0.098		

Formal Description The sensitivity analysis algorithm returns a set of parameters and their sensitivity, according to:

$$Sens(C_B) = \{(p, min(SensP(C_B, p)) : p \in P\} \tag{1}$$

with P being the set of parameters under investigation and C_B being a reference parameter configuration comprising the standard values for each parameter, determined in previous experiments. $min(S)$ calculates the minimum of a set S of values and is applied to $SensP(C_B, p)$, which contains the relative change of a parameter p required to get a relative deviation of 10% in the simulation analysis results. This is illustrated in the following formula:

$$SensP(C_B, p) = \left\{ \frac{|arg_p(C_B) - arg_p(C_p)|}{arg_p(C_B)} : \frac{|R(C_B) - R(C_p)|}{R(C_B)} > 0.1 \right\} \tag{2}$$

with C_P being a parameter configuration, which differs from C_B only in the value of parameter p. $arg_p(C)$ returns the value of parameter p of a given configuration C. $R(C)$ returns the simulation analysis results (i.e., the results obtained simulating a configuration and analyzing its output) of configuration C. It

basically repeats simulation runs and calculates the arithmetic mean of their results, to gain a given confidence:

$$R(C) = \Sigma_{i=1}^{n} \frac{X_i(C)}{n} \quad (3)$$

with $X_i(C)$ being the simulation result of run i with configuration C. The confidence is gained by a two stage approach [1] (page 71), where an initial sample of simulation runs is executed and the overall amount of required runs n for the given configuration is calculated by:

$$n = \left(\frac{Z_{0.95} \cdot S_X}{e \cdot \overline{X}} \right)^2 \quad (4)$$

with $Z_{0.95}$ being the 0.95-quantil of the standard normal distribution (to achieve 95 percent confidence), $\overline{X}$ being the sample mean of the simulation run results, S_X being the according sample standard deviation, and e being the allowed, relative error tolerance (which we set to 0.05). Note, that the focus of this approach is on achieving a given confidence in the simulation results, not in executing a given count of replication. Therefore, and due to the fact that we calculate the required amount of replications for each of the many executed model configuration, presenting all calculated replication counts is not necessary nor feasible for this paper.

The simulation results in equation 3 are calculated by $X_i(C) = ED(Y_i^{D(C)}, Y^W)$, where $Y_i^{D(C)} = y_{i,0}^{D(C)}, ..., y_{i,m}^{D(C)}$ is the trajectory generated by executing a simulation run of configuration C and $Y^W = y_0^W, ..., y_m^W$ being the reference trajectory we achieved in our wet-lab experiments. We compared both trajectories by calculating the euclidean distance [2] (page 94):

$$ED(Y^{D(C)}, Y^W) = \sqrt{\Sigma_{j=0}^{m} (y_{i,j}^{D(C)} - y_i^W)^2} \quad (5)$$

For the comparison, we had to adapt the experimental data, as it was based on concentrations, while the trajectories created by simulation where based on molecule numbers. Hence, we calculated the molecule numbers of the sub sequent observation points by multiplying the initial molecule count with the ratio between initial concentration and the concentration at the point:

$$y_i^W = y_0^W * c_i / c_0 \quad (6)$$

The concrete value are depicted in Table S2.

Table S2. Reference trajectory with molecule numbers.

time (h)	molecule number
0	5345
0.5	7430
1	7857
2	5452
8	8178
12	9193

References

1. Asmussen S, Glynn PW (2007) Stochastic Simulation. Springer.

2. Deza E, Deza MM (2009) Encyclopedia of Distances. Springer.
